# Supplementary material for: Role of Ezrin/Radixin/Moesin in the Surface Localization of Programmed Cell Death Ligand-1 in Human Colon Adenocarcinoma LS180 Cells
Source: Pharmaceuticals (Basel). 2021 Aug 28;14(9):864. doi: 10.3390/ph14090864 (PMC8467328; doi:10.3390/ph14090864)
Supplement: Supplementary file 1 [file pharmaceuticals-14-00864-s001.zip › pharmaceuticals-1334331-supplementary_Author Rev_New.pdf]

pharmaceuticals

MDPI

Supplementary Materials

Role of Ezrin/Radixin/Moesin in the Surface Localization of Programmed Cell Death Ligand-1 in Human Colon Adenocarcinoma LS180 Cells

Takuro Kobori[1], Chihiro Tanaka[1], Mayuka Tameishi[1], Yoko Urashima[1], Takuya Ito[2], and Tokio Obata[1,*]

[1] Laboratory of Clinical Pharmaceutics, Faculty of Pharmacy, Osaka Ohtani University, Tondabayashi, Osaka 584-8540, Japan; koboritaku@osaka-ohtani.ac.jp (T.K.); u4117078@osaka-ohtani.ac.jp (C.T.); u4117083@osaka-ohtani.ac.jp (M.T.); urasiyo@osaka-ohtani.ac.jp (Y.U.)
[2] Laboratory of Natural Medicines, Faculty of Pharmacy, Osaka Ohtani University, Tondabayashi, Osaka 584-8540, Japan; itoutaku@osaka-ohtani.ac.jp (T.I.)
* Correspondence: obatatoki@osaka-ohtani.ac.jp (T.O.); +81-721-24-9371 (T.O.)

Gene Silencing of Moesin Upregulates the mRNA Expression Levels of Proinflammatory Cytokines in LS180 Cells

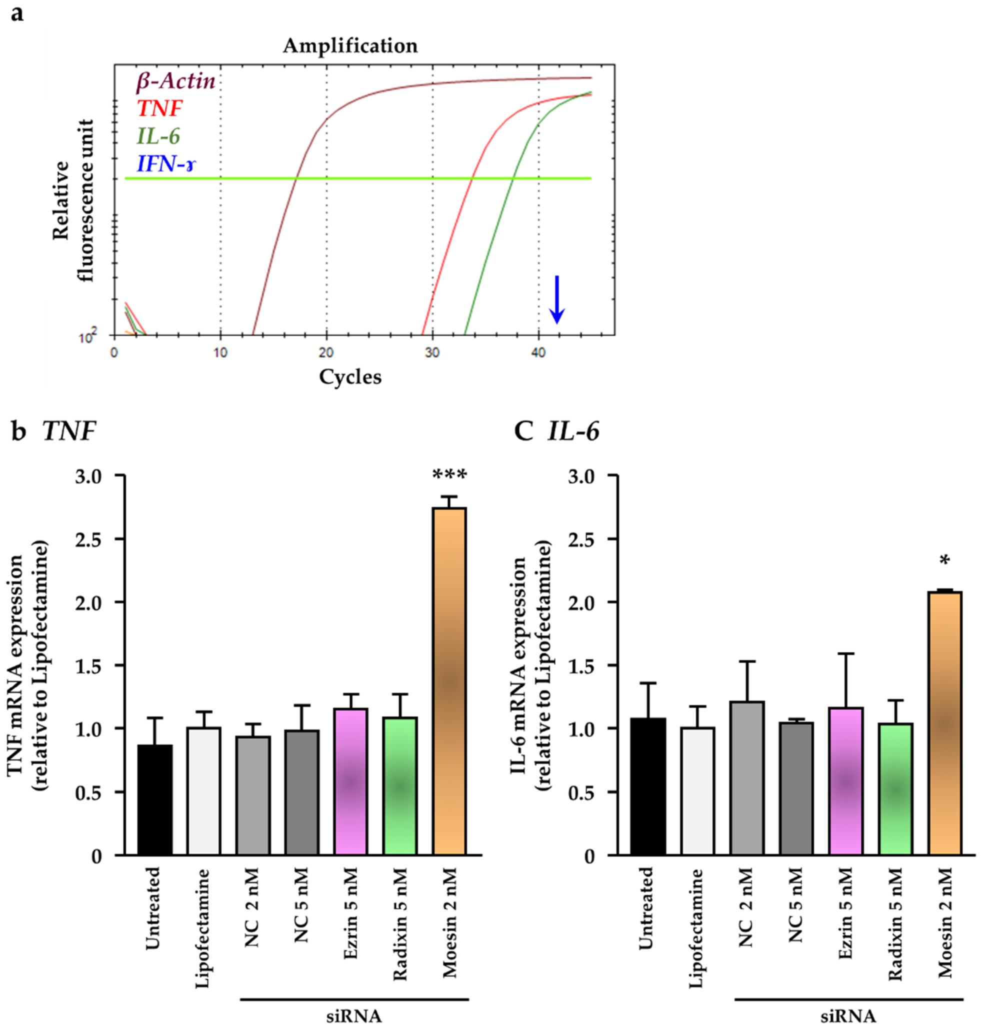

Figure S1. Gene Silencing of Moesin Upregulates the mRNA Expression Levels of Proinflammatory Cytokines in LS180 Cells. Cells were incubated with the transfection medium (Untreated), transfection reagent (Lipofectamine), nontargeting control (NC) siRNA, and specific siRNAs for ezrin, radixin, or moesin and then cultured for 3 days. (**a**) Representative amplification curves of β-Actin (brown line), tumor necrosis factor (TNF) (red line), interleukin (IL)-6 (green line), and interferon (IFN)-ϒ (blue line; undetectable) mRNA expressions in the Untreated cells as determined by real-time quantitative reverse transcription-polymerase chain reaction. Gene expression levels of (**b**) TNF and (**c**) IL-6 mRNA normalized with β-actin in cells treated with each siRNA relative to that in cells treated with the transfection reagent alone. n = 3, ****p* < 0.001, **p* < 0.05 vs. Lipofectamine. All data were expressed as the mean ± SEM and analyzed by one-way ANOVA followed by Dunnett's test.

Changes in the Cell Viability of LS180 cells by Treatment with ERM siRNAs at the Concentrations of 2 and 5 nM

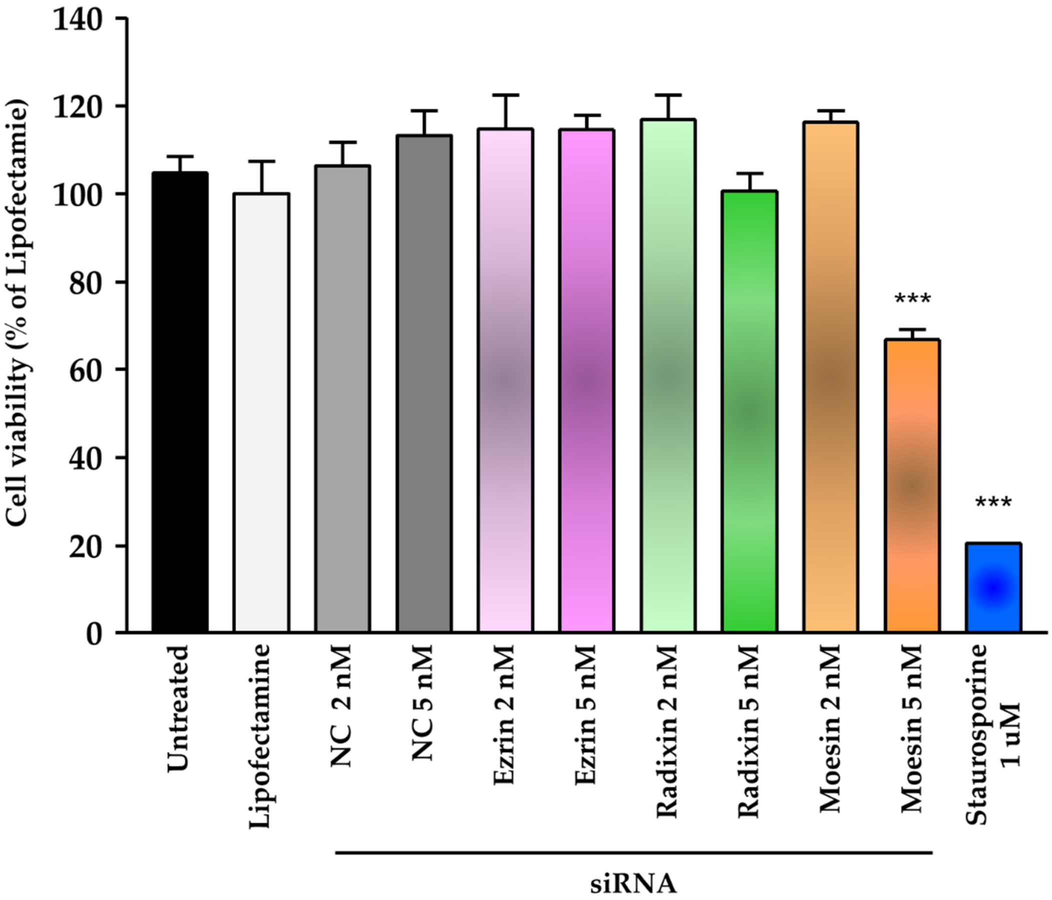

Figure S2. Changes in the Cell Viability of LS180 cells by Treatment with ERM siRNAs at the Concentrations of 2 and 5 nM. Cells were treated with the transfection medium (Untreated), transfection reagent (Lipofectamine), nontargeting control (NC) siRNA, and specific siRNA for ezrin, raidixn, or moesin and then incubated for 3 days. The concentrations of all siRNAs used were 2 and 5 nM. Cell viability was assessed with the PrestoBlue cell viability reagent. Staurosporine 1 μM was used as a positive control for inducing cell death. n = 3, ***$p < 0.001$ vs. Lipofectamine. All data were expressed as the mean ± SEM and analyzed by one-way ANOVA followed by Dunnett's test.

Materials and Methods for Figure S2

Cell Viability Assay

LS180 cells were cultured until 70–80% confluent, and then were seeded at a density of 5.0 × 10^3 cells/well in 96-well cell culture plates (Thermo Fisher Scientific, Tokyo, Japan). The cultures were incubated overnight at 37°C in a humidified atmosphere with 5% CO_2 to allow for attachment. Silencer Select small interfering RNA (siRNA) targeting human ezrin, radixin, or moesin, and Silencer Select Negative Control siRNA (Thermo Fisher Scientific) were diluted with Opti-MEM (Thermo Fisher Scientific). The cells were then transfected with each siRNA (2 and 5 nM) using the Lipofectamine RNAiMAX Transfection Reagent (Thermo Fisher Scientific) at the volume of 0.2 μL/well. At the same time, cells were treated with staurosporine 1 μM (Merck, Darmstadt, Germany) as a positive control for inducing cell death. After treatment of cells with siRNAs or staurosporine, cells were continuously cultured for 3 days without exchanging medium. Thereafter, 10 μL/well of a commercial PrestoBlue Cell Viability Reagent (Thermo Fisher Scientific, Tokyo, Japan) was added directly to the wells containing 100 μL of the complete growth medium and the cells were incubated for 10 min at 37°C in a humidified atmosphere with 5% CO_2 protected from direct light. Subsequently, fluorescence signals were detected at a wavelength of 560 nm (excitation) and 590 nm (emission) using a Synergy HTX Multi-Mode Microplate Reader (BioTek Instrument, Winooski, VT, USA). PrestoBlue is a new resazurin-based reagent

to assess cell viability and cytotoxicity with higher sensitivity than 3-(4,5-dimethyl-2-thiazolyl)-2,5-diphenyl-2H-tetrazolium bromide (MTT), and comparable with that of Alamar Blue [1-4].

References for Figure S2

1. Xu, M.; McCanna, D. J.; Sivak, J. G., Use of the viability reagent PrestoBlue in comparison with alamarBlue and MTT to assess the viability of human corneal epithelial cells. *J. Pharmacol. Toxicol. Methods* **2015,** 71, 1-7.
2. Lall, N.; Henley-Smith, C. J.; De Canha, M. N.; Oosthuizen, C. B.; Berrington, D., Viability Reagent, PrestoBlue, in Comparison with Other Available Reagents, Utilized in Cytotoxicity and Antimicrobial Assays. *Int. J. Microbiol.* **2013,** 2013, 420601.
3. Stockert, J. C.; Horobin, R. W.; Colombo, L. L.; Blazquez-Castro, A., Tetrazolium salts and formazan products in Cell Biology: Viability assessment, fluorescence imaging, and labeling perspectives. *Acta Histochem.* **2018,** 120, 159-167.
4. Boncler, M.; Rozalski, M.; Krajewska, U.; Podsedek, A.; Watala, C., Comparison of PrestoBlue and MTT assays of cellular viability in the assessment of anti-proliferative effects of plant extracts on human endothelial cells. *J. Pharmacol. Toxicol. Methods* **2014,** 69, 9-16.

Original Western Blotting Images of Target Proteins in LS180 Cells

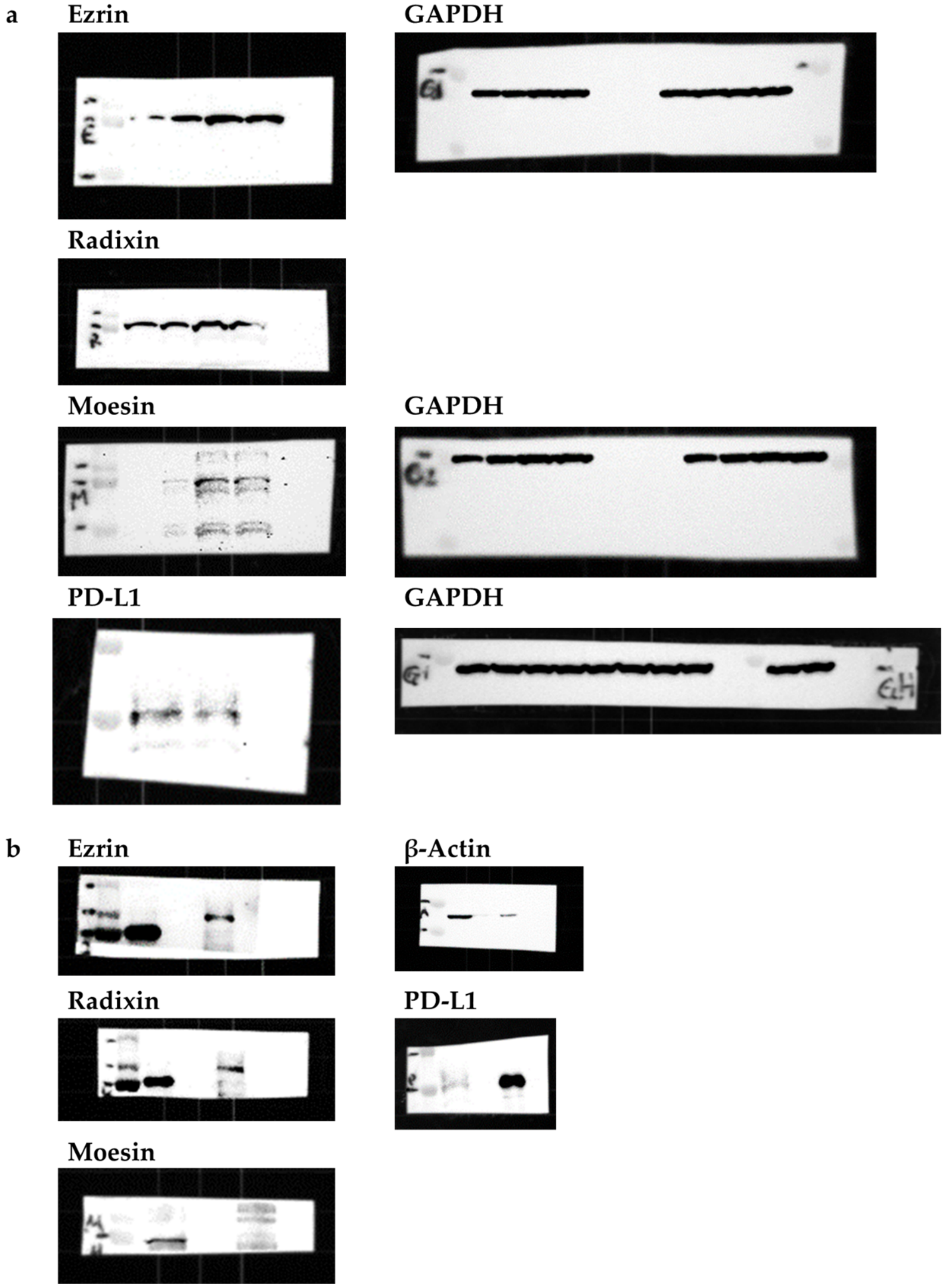

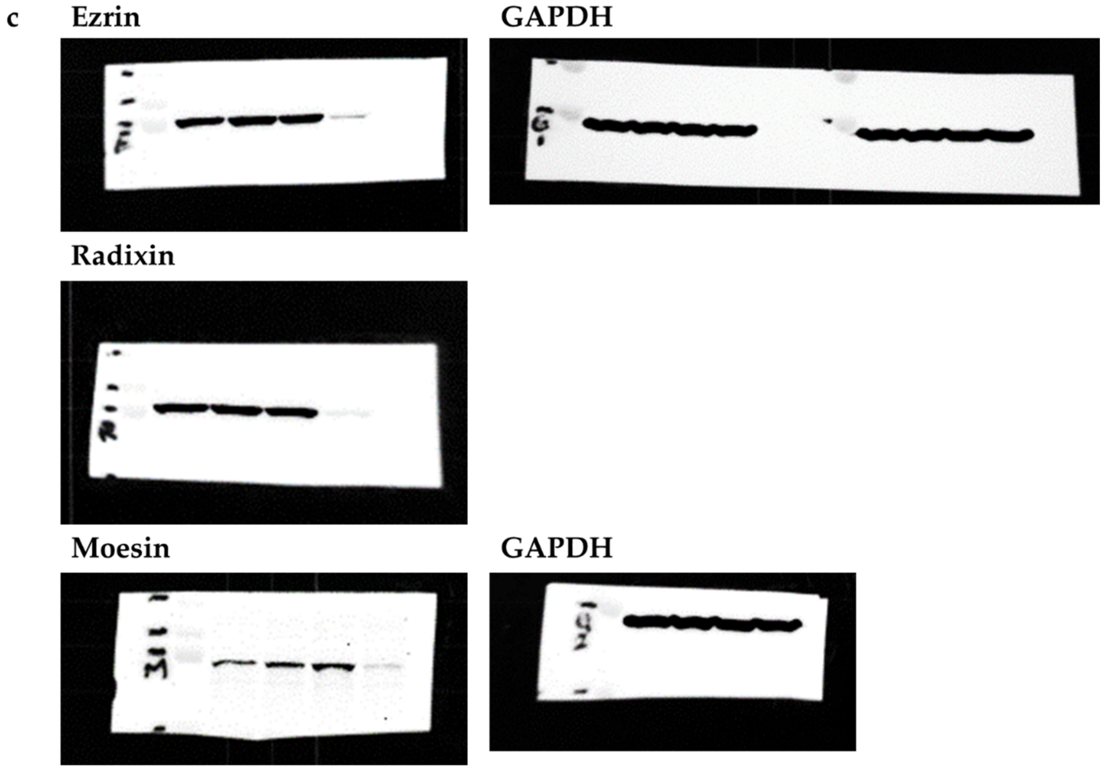

Figure S3. Original Western Blotting Images of Target Proteins in LS180 Cells. **(a)** The original western blotting membrane to check the expression pattern of ezrin, radixin, moesin, and programmed cell death ligand-1 (PD-L1) at protein levels shown in Figure 1e. **(b)** The original western blotting membrane to detect the protein expressions of PD-L1, ezrin, radixin, and moesin as well as β-actin in the whole cell lysates (input) and that immunoprecipitated (IP) with a control antibody or an anti-PD-L1 antibody as indicated in Figure 4. **(c)** The original western blotting membrane to measure the protein expression levels of ezrin, radixin, and moesin in addition to glyceraldehyde-3-phosphate dehydrogenase (GAPDH) as shown in Figure 5d-f.
